# Monitoring central nervous system tumour metabolism using cerebrospinal fluid

**DOI:** 10.3389/fonc.2024.1389529

**Published:** 2024-12-05

**Authors:** Alison Whitby, Madhumita Dandapani

**Affiliations:** Children’s Brain Tumour Research Centre, Biodiscovery Institute, School of Medicine, University of Nottingham, Nottingham, United Kingdom

**Keywords:** cerebrospinal fluid, metabolomics, brain cancer, recurrence, monitoring, biomarkers

## Abstract

Central nervous system (CNS) tumours are the most common cancer cause of death in under 40s in the UK, largely because they persist and recur and sometimes metastasise during treatment. Therefore, longitudinal monitoring of patients during and following treatment must be undertaken to understand the course of the disease and alter treatment plans reactively. This monitoring must be specific, sensitive, rapid, low cost, simple, and accepted by the patient. Cerebrospinal fluid (CSF) examination obtained following lumbar puncture, already a routine part of treatment in paediatric cases, could be better utilised with improved biomarkers. In this review, we discuss the potential for metabolites in the CSF to be used as biomarkers of CNS tumour remission, progression, response to drugs, recurrence and metastasis. We confer the clinical benefits and risks of this approach and conclude that there are many potential advantages over other tests and the required instrumentation is already present in UK hospitals. On the other hand, the approach needs more research investment to find more metabolite biomarkers, better understand their relation to the tumour, and validate those biomarkers in a standardised assay in order for the assay to become a clinical reality.

## 1 Introduction

Central nervous system (CNS) tumours are cancers which originate in the brain and spinal cord, either as localized or metastatic lesions, and range from benign tumours that cause disability and can still be life threatening (*e.g*. craniopharyngioma), to high grade, progressive cancers (*e.g*. medulloblastoma). These are collectively the biggest cancer killer of people under the age of 40 in the UK (1). This is largely due to the difficulty of total tumour resection and finding targeted drugs that pass through the blood brain barrier (BBB), as well as the persistent, recurrent nature of these tumours and difficulty in monitoring early recurrence (2–5). Recurrence comes about through persistent minimal residual disease (MRD), where tumour cells remain after treatment without signs or symptoms, as tumours often recur at or near the original site, and it is important to detect and treat these earlier.

**Figure 1:**
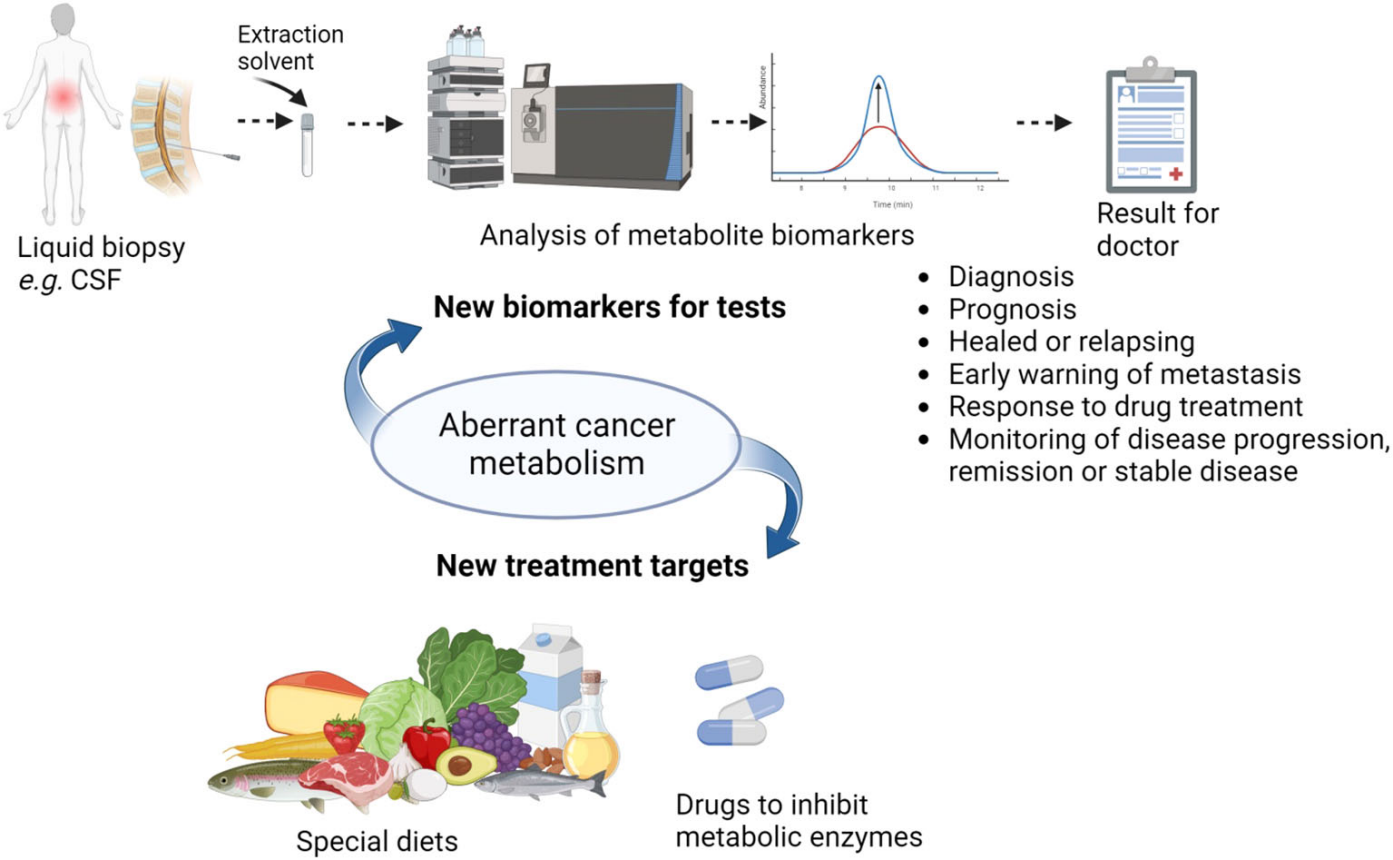
The potential of aberrant cancer metabolism to impact health outcomes in CNS cancers and the many potential uses of CSF metabolite biomarkers (Created with BioRender.com).

CNS tumours have aberrant cancer metabolism, which could be harnessed clinically in a number of ways. Firstly, it potentially offers a novel means to treat cancers [for example by the ketogenic diet (6, 7), or by therapeutic arginine depletion through consuming arginase to deplete extracellular arginine, to counter auxotrophism (8, 9)] (**Figure 1**). Secondly, it offers a potential means to monitor the presence of the cancer during and following treatment (*i.e.* offers biomarkers), in order to detect recurrence early on and expedite second treatment. For example, magnetic resonance spectroscopy (MRS) of brain tumours can be used to identify tumours by the levels of choline and N-acetyl aspartate (10) and found tumour metabolite profiles at diagnosis remain highly similar at first recurrence, helping recurrence diagnosis (11). Additionally, ^11^C-methionine-positron emission tomography could detect glioma recurrence sensitively and specifically (12). The ability of metabolism to pinpoint cancer recurrence has been confirmed in tumors outside the central nervous system using MRS or serum biopsy, such as in prostate cancer (13), breast cancer (14), myeloma (15) and colorectal cancer (16). These longitudinal studies of cancer following treatment commencement must distinguish between cancer and drug-induced metabolic changes (17). Metabolite levels at the time of primary surgery might even predict the likelihood of tumour recurrence (18). Thirdly, side effects of cancer treatment could be monitored by their influence on metabolism and alleviated early (19).

Metabolomics is the study of all metabolites (the metabolome) within cells, a tissue or an organism. This review will outline metabolic reprogramming in cancer and apply to CNS cancers, explain how these metabolites can be detected in the cerebrospinal fluid (CSF), whether those metabolites correlate with those in the tumour or Magnetic Resonance Imaging/Spectroscopy (MRI/MRS) images, and synergy with monitoring drug delivery. The review will especially focus on clinical applicability of CSF sampling and metabolite biomarker tests as well as the challenges and future directions.

## 2 Introduction to metabolic reprogramming in tumours especially CNS tumours

Ever since Otto Warburg discovered aerobic glycolysis in tumours, metabolic changes in tumours have been investigated and new knowledge gained, so reprogramming energy metabolism is now a hallmark of cancer (20). These metabolic changes are varied, and many are specific to a certain cancer, so that they could perhaps be targeted by personalised anti-cancer drugs or used in diagnosis (21, 22). Many examples of aberrant metabolism in brain tumours are known. One-carbon metabolism, determined by the rate of diversion of carbons into polyamine metabolism, is altered in ependymoma, and affects the epigenome (23). Carbohydrate metabolism is altered in glioma, with accumulation of glycolytic intermediates leading to accumulation of pentose phosphate pathway intermediates, showing anabolic metabolism in grade IV tumours (24). Additionally, different monosaccharides are incorporated into glycans (mannose vs sialic acid), affecting extravasation of bone marrow-derived human mesenchymal stem cells and antioxidant status (25). Glycerophospholipid metabolism is augmented in medulloblastoma as it has a four times higher level of phosphocholine than does ependymoma or pilocytic astrocytoma, and a phosphocholine/glycerophosphocholine ratio of 8.6 compared to 0.8-1 for the other cancers and 0.4 for normal tissue (26). Glycerophospholipid metabolism is involved in synthesising membranes and intracellular signaling. In some gliomas, one population of cells is in aerobic glycolysis while the other uses the secreted lactate through the citric acid cycle, working in symbiosis (20, 27, 28). Brain tumours can be metabolically heterogeneous, which complicates the interpretation of results gained from sampling the tumour from a single region (29–33). Therefore, monitoring tumour metabolism through a systemic biofluid such as blood or CSF should present an average of the metabolome of the whole tumour and be an accessible way to monitor remission and recurrence.

## 3 Advantages and limitations of CSF liquid biopsy in brain cancer

Advantages of using CSF to diagnose or monitor brain cancer are that it is minimally invasive compared to surgical tumour biopsy, it is quicker, it requires smaller specialist hospital space, and it is cheaper (34, 35). CSF analysis may also be more specific than an MRI scan as MRI is not always helpful in working out what an abnormality is and distinguishing between treatment reaction and recurrence, though it is useful in defining location (10, 11, 36, 37). CSF analysis is especially useful in cases where the tumour cannot be biopsied such as DIPG (38) and also in long term monitoring of patients who have received surgery but could recur (39, 40). However, using CSF does have some drawbacks, for example, it could incur complications such as headache, herniation, back pain, infection and bleeding (some of which are potential complications of surgery too), which are distressing to the patient and could contaminate the sample (35). As lumbar puncture is minimally invasive, the risk of adverse effects must be balanced against the degree of benefit to the patient, in terms of the increased likelihood of survival following either earlier diagnosis of progression/recurrence or a more accurate prognosis. The benefit must outweigh the risks. Nevertheless, although lumbar puncture is more challenging and less pleasant than phlebotomy, it is often done as part of standard clinical testing in paediatric patients and so samples are available for pathology (41, 42). CSF samples in these patients are normally taken at 14 days post-surgery which is before adjuvant therapy, and at any scan that shows recurrence or metastasis. The safest and purest time to collect CSF is post-operatively (43) however it should be close to the time of operation as the presence of a tumour may be important (34). Therefore the idea of taking the sample intraoperatively and centrifuging out contaminating cells is suggestive of gaining a good baseline metabolome of the cancer. Certainly, glioblastoma CSF samples pre-treatment and post-treatment were different, with treatments including chemotherapy, radiation therapy, and/or tumor-treating field, so baseline samples should be pre-adjuvant treatment (44).

## 4 Advantages of measuring CSF circulating biomarkers

Measuring CSF circulating biomarkers is likely to be better than CSF cytology, as CSF cytology produces false negatives because of its low sensitivity and is qualitative (35). Sensitivity increased from 69% to 92% when diagnosis was based on the NMR spectrum of CSF circulating metabolites rather than on CSF cytology, with only a small decrease of 4% in specificity (36). CSF circulating biomarkers are independent from circulating tumour cells and so perhaps leak from the tumour itself, making them interesting for understanding the tumour (35).

The CSF is likely to be better for monitoring circulating biomarkers of brain cancers than the blood, because of the relative impermeability of the BBB and the proximity of brain tumours (especially ependymoma and leptomeningeal disease, which grow around the ventricles in the brain) to CSF [(35, 40, 45), see also (46)]. Circulating tumour DNA (ctDNA) exists at higher concentrations in CSF than in plasma because of the BBB (47). Furthermore, levels of peripheral metabolites in blood are not always correlated to central metabolites in CSF and though differentially abundant in blood, may not be biologically relevant (41). Levels of metabolites in plasma and CSF usually correlated in high grade glioma patients, making CSF analysis as good as more well-established blood analysis in this case, possibly due to a leaky BBB in samples taken on the first day of hospital admission (48, 49). Contrastingly, in initially dementia-free middle-aged adults enriched for parental history of Alzheimer’s disease (AD), correlations between levels of plasma and CSF metabolites varied widely but typically had low correlations (50). Various reasons were suggested for this, including many metabolites not crossing the blood-brain barrier, most plasma metabolites being unrepresentative of certain metabolic changes occurring in the brain, and blood and CSF samples being taken on different days (on average within 27 days). It was concluded that CSF metabolites give a more useful indication of AD risk. Although there was overlap in the plasma and CSF metabolomes in children with b-cell acute lymphoblastic leukemia, with 90% of the CSF metabolites also detected in plasma, the median Spearman rank correlation between metabolite levels in each fluid was only 0.37 (51). Fifty nine percent of metabolites had weak correlations, whereas 19% had moderate correlations and 20% had strong correlations, with xenobiotics (metabolites not arising from the tumour or body) overrepresented in the strong correlates. Clearly, one would expect metabolites with biological significance to have strong correlation, but in order to know which metabolites in blood have biological significance, both CSF and blood metabolites must be analysed. Xenobiotics are unlikely to be of use as biomarkers since they are likely to vary in level from person to person, as levels are due to environmental factors. Therefore, comparative studies of CSF and blood are needed to develop blood biomarkers that are present in both biofluids (bridging biomarkers), which are definitely reflective of central changes, before blood can be used for monitoring CNS cancers (41).

## 5 Possibilities of ctDNA, miRNA, proteins and metabolites as CSF circulating biomarkers

Circulating tumour DNA (ctDNA) genomic analysis can be undertaken by next-generation sequencing at a reasonable cost and higher sensitivity than cytology (35). In a systematic review including 19 studies of genomic biomarkers, for example, CSF genome copy number variations have been used as a marker for MRD in medulloblastoma, with half of patients with complete radiographic response to treatment then showing MRD through CSF biomarker presence three months before radiography showed recurrence (52). Nevertheless, ctDNA is not always detected in CSF, and is more likely to be detected if the tumour is next to the ventricle (5, 52, 53). Technical sensitivity can also be an issue hampering ctDNA detection (35). As miRNAs and mRNAs in CSF are in low abundance and labile, most studies employed targeted methods to profile them rather than systematically probing for biomarkers (45). miRNAs are however stable inside extracellular vesicles and can be isolated from them (35). Some miRNAs are specific for brain metastases of cancers and some are upregulated in one brain cancer and downregulated in another (35). However, more work needs to be done on CSF miRNA extraction protocols before miRNA can be used in the clinic and we need to understand whether the environment affects the CSF miRNA profile. The assays may not be sensitive enough. Additionally, we do not know which genes all the miRNAs affect and yet it is important to have a scientific rationale for a clinical treatment. More commonly found by the systematic review, with 23 papers, were studies analysing protein biomarkers in CSF (52). For example beta-human chorionic gonadotropin (ß-HCG) for intracranial germ cell tumours, polysialic-neural cell adhesion molecule (PSA-NCAM) for medulloblastoma, and cyclophillin A and dimethylarginase 1 for DIPG (52). Protein biomarkers are promising, but drawbacks are that proteins are hard to identify by proteomics, which is expensive and time-consuming, and enzyme linked immunosorbent assay (ELISA) requires a well-defined, highly-specific antibody (52).

**Figure 2:**
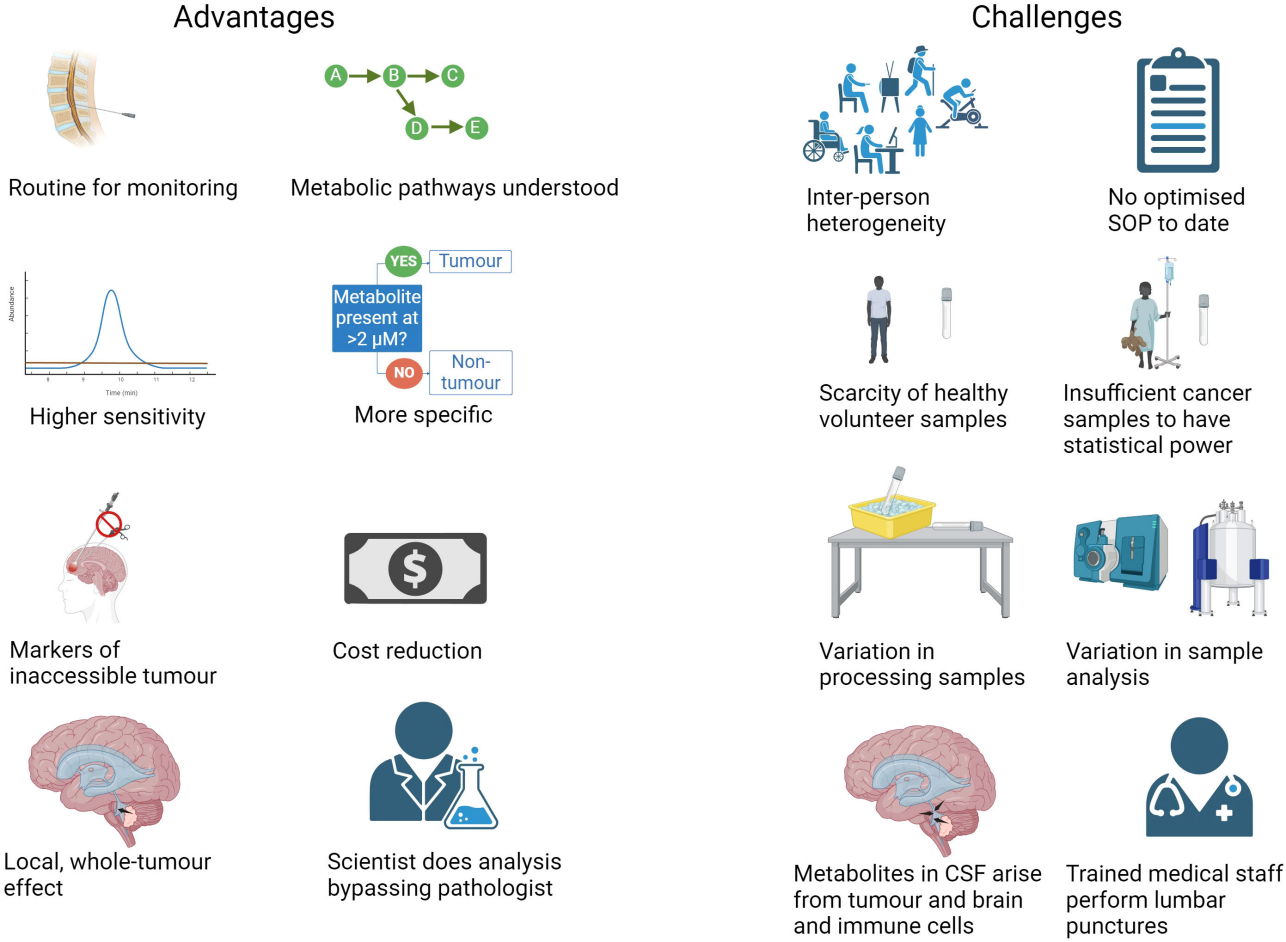
Advantages and challenges of using CSF metabolite biomarkers to diagnose and monitor CNS tumours (Created with BioRender.com).

On the other hand, most human metabolites have been mapped onto inter-linking metabolic pathways with enzymes catalyzing reactions known, and so there is scientific rationale for metabolic biomarkers of cancer, especially when more than one metabolite in a pathway is increased or decreased in abundance in a cancer (**Figure 2**). Even uncommon metabolites that are oncometabolites have had their reactions and enzymes characterized, such as D-2-hydroxyglutarate and morphine (54–56). Compared to ctDNA and miRNA, metabolites in CSF can be detected more cheaply per sample at a lower threshold, not requiring amplification (though sometimes samples are concentrated), by automation using mass spectrometry (MS) or nuclear magnetic resonance (NMR) spectroscopy, and when compared to CSF cytology and MRI scans, metabolites can be detected economically and more quickly, bypassing the need for the time of expert pathologists or radiologists (35, 36, 57). The conservation of core metabolites across species enables samples from different species to be compared to one another *e.g*., in research comparing animal experiments to human study. The metabolites identified in CSF have been curated and number 468 so far (58).

Some of the metabolites altered in abundance in the CSF of brain tumour patients compared to non-cancer patients or healthy volunteers were amino acids (proline, glutamine, serine, lysine, methionine, tryptophan, tyrosine, beta-alanine, leucine, isoleucine, aspartic acid, 3-methylhistidine, anserine and histidine), lipids and lipid catabolites (2-ketohaxanoic, phytosphingosine, oleic acid, myristoleic acid, PE 34:1, farnesyl diphosphate, 12,13-DiHOME (a beta-oxidation promoting lipid hormone), total triacylglycerols, acetylcarnitine, propionylcarnitine, butyrylcarnitine, isobutyrylcarnitine, 2-methylbutyrylcarnitine, deoxycarnitine) (33, 34, 43–45, 57, 59–61). Also, there were pyrimidines (dihydroorotate, orotate, dTMP, uridine) and purines (purine, xanthine, 7-methylguanosine, adenine, hypoxanthine, 1-methyladenosine). Additionally, central carbon metabolites (isocitrate, malic acid, succinate, fumarate, α-ketoglutarate, hydroxypyruvate, lactate), lysine catabolites (aminoadipic acid, pipecolic acid, N6-acetyl-l-lysine), urea cycle metabolites (ornithine, citrulline, arginine), vitamins (pantothenic acid, flavin adenine dinucleotide (FAD), dehydroascorbic acid), and a bacterial metabolite (shikimate) were also differentially abundant. The common brain metabolites (taurine, N-acetyl aspartic acid, myo-inositol, γ-aminobutyric acid (GABA) and its precursor aminobutanal, choline), one carbon metabolites (betaine, betaine aldehyde, S-(5′-adenosyl)-L-methionine) and those in creatine anabolism (guanidinoacetic acid and creatine) rounded off the main metabolites altered in abundance (**Table 1**). It was possible to distinguish medulloblastoma (MB) from healthy controls but not distinguish MB subgroups by CSF metabolome, so CSF metabolomics is a broad brush stroke to be used for monitoring disease recurrence and not fine detail diagnosis (45). On the other hand, the genetic mutations in glioblastoma could be distinguished by CSF metabolite levels (44). The metabolite biomarkers in CSF have many potential uses (**Figure 1**). Firstly, they can distinguish cancer patients from healthy people, sometimes quantitatively, and so aid primary diagnosis or diagnosis of recurrence (33, 44, 45, 57). This is especially of use in diagnosing when the tumour is not resected due to location, and so not available for histology, as explained earlier for DIPG (*vide infra*). Metabolite levels sometimes correlate with patient survival and so could aid prognosis (33, 44). Levels of metabolite biomarkers in surveillance tests can be compared to the level at diagnosis, for an individual patient, either to detect recurrence as soon as it appears, since in MRS scans metabolite levels are known to be the same in the primary and recurrent paediatric brain tumour (11), or to distinguish progression to a higher stage from remission and stable disease by targeted metabolomics, as seen in LC-MS/MS of serum samples in colorectal cancer (16) and untargeted LC-MS of CSF samples in leukaemia tracking CNS involvement (62). An untargeted CSF study showed glioma progression by metastasis or malignant transformation to higher grade (34). To potentially detect metastasis early, it is of interest to monitor the CSF metabolome of medulloblastoma and other metastasising brain cancers, because these cancer cells utilise nutrients in the CSF when metastasising through the CSF (63). The perturbed metabolism in tumours may lead to new treatment targets [*e.g*. IDH1 mutant inhibitor or depleting an intermediate with autophagy (34)]. Finally, the CSF metabolome could be used in monitoring response to drug treatment (64, 65).

**Table 1:** Summary CSF metabolomics studies of brain tumour patients.

Population	Metabolomics platform	Metabolite abundance changes in disease vs control	Study
10 malignant gliomas vs 7 controls	LC-MS targeted	↑: Proline, methionine, lysine, phenylalanine, serine, glutamine, acetylcarnitine, dihydroorotate, orotate, dTMP, purine, 7-methylguanosine, aminoadipic acid, N6-acetyl-L-lysine, taurine, biotin, gluconolactone, indole 3-carboxylic acid, 2,3-dihydroxybenzoic acid, phenyllactic acid, phenylpropiolic acid, shikimate, atrolactic acid, N-acetyl-glutamine, thiamine, 2-ketohaxanoic acid, S-methyl-5-thioadenosine. ↓: Adenine, hypoxanthine, isocitrate, myo-inositol, ribose-phosphate, nicotinamide, glucose 1-phosphate, 2-hydroxy,2-methylbutanedioic acid.	(33)
14 grade IV vs 8 grade III vs 2 grade I and 8 grade II (in one group) gliomas (preoperative samples). IDH mutant vs IDH-wild type in grades I-III.	GC-MS targeted and untargeted	↑: Citric, isocitric acids (grades IV vs grades I-II and III). ↑: Citric, isocitric, lactic acids; ↓: Pyruvate + oxaloacetic acid (combined signal) (mutant vs wild type).	(59)
26 lung adenocarcinomas with signs of leptomeningeal carcinomatosis vs 41 controls	NMR spectroscopy untargeted	↑: Citrate, alanine, lactate. ↓: Creatine, myo-inositol.	(36)
23 patients with a range of tumour types (*i.e.*, glioma IDH-mutant (G-mut, n=4 with 13 samples), glioma-IDH wild type (G-WT, n=7), metastatic lung cancer (LM, n=7) and metastatic breast cancer (BM, n=5)) vs 8 control patients (NT)	LC-MS targeted (3x chromatography)	↑: Malic acid, succinate, aminobutyric acid, pipecolic acid, aminoadipic acid, creatine, betaine, guanidinoacetic acid, glucosamine, proline, aspartic acid, N-acetyl aspartic acid, taurine, butryl carnitine; ↓: Oleic acid, myristoleic acid (T vs NT). ↑: Glycine-leucine, glucosamine/galactosamine, malic acid, 3-phosphoglycerate, arginino-succinate, 3PG and 2PG; ↓: Alanine (LM vs NT). ↑: Guanidinoacetic acid, betaine, glucosamine/galactosamine, ornithine, methylcysteine, ethonalamine, aminophosphovaleric acid, 3-phosphoglycerate, 3PG and 2PG, 5-methyl-5-thioadenosine, cysteine, quinic acid, lactate, glutamic acid, 3-hydroxykyurenine, amino adipic acid, cystathionine, malic acid, succinate, phosphoenolpyruvate (BM vs NT). ↑: Acetylcarnitine, shikimate, aminobutyric acid, N-acetylaspartic acid, FAD (G-WT vs NT). ↑: Succinate, malic acid, lactic acid, aspartic acid, N-acetylaspartic acid, citrulline (G-mut vs NT). ↑: 1-Methyl-histidine, arginine, asparagine, N-acetylputrescine, malonate, betaine aldehyde, pantothenic acid; ↓: 1-methyl tryptophan, succinic acid semialdehyde (G-mut vs G-WT).	(57)
Primary central nervous system lymphomas (PCNSL, n = 59), secondary central nervous system involvement of systemic lymphomas (SCNSL, n = 11), lung adenocarcinomas with brain metastases (MBT, n = 34), and lung adenocarcinomas without brain metastases (NMBT, n = 23) vs nontumorous brain diseases controls (NT, n = 36)	LC-MS untargeted	↑: Phytosphingosine, cystine, glutamine, 5-aminoimidazole, butyrylcarnitine (PCNSL vs NT). ↑: Phytosphingosine, 1-methyladenosine, cystine, 5-aminoimidazole, dehydroascorbic acid, prolyl-threonine (MBT vs NT). ↑: Acetylcarnitine, butyrylcarnitine; ↓: Inositol phosphate, succinyladenosine, hypoxanthine, creatinine, homocysteine, valyl-methionine (MBT vs NMBT). ↑: Glutamine, prolyl-threonine, dehydroascorbic acid, 5-aminoimidazole, uracil, cystine, phytosphingosine; ↓: Isovaleric acid (T vs NT).	(60)
8 recurrent paediatric medulloblastomas (before therapy), 7 controls	LC-MS targeted (3x chromatography)	↑: Tryptophan, tyrosine, methionine, lysine, beta-alanine, serine, 12,13-DiHOME, a molecule isobaric to docosapentaenoic acid. ↓: Proline, leucine, isoleucine, 3-methylhistidine, histidine, PE 34:1.	(61)
25 gliomas (with 7 multi-time samples coming from 3 patients giving total 32 samples, some of whom have leptomeningeal metastasis (LM)), 10 controls, 7 non-glial brain tumours (medulloblastoma (MB)).	LC-MS untargeted	↑: 99 low mass ions (Glioma vs control). ↑: Glycol aldehyde, glyceric acid, acetic acid; ↓: Sphingolipid metabolites *e.g*. sphingosine, ceramide (Grade IV vs grade III). ↑: Carnitine, glycerophosphate; phosphatidylinositol, phosphatidylserine; ↓: Carboxylic acid or pteridine (Grade IV vs MB). ↑: Ethanone, lactone, norepinephrine, hydroxydopamine, pyruvaldehyde, cysteine; ↓: Diacylglycerol, phosphatidylserine (LM+ vs LM- within grade III). ↑: Acetic acid, glycolaldehyde, pyruvate aldehyde; ↓: Diacylglycerol, phosphatidylserine (LM+ vs LM- within grade IV).	(34)
83 CSF samples from 45 medulloblastoma patients with documented MRI and CSF cytology results at the time of sampling for LM (either ++ n=8, +- n=20, -+ n=8 or – n=47)	LC-MS untargeted	↑: 23 putatively identified - vitamins B3, B12 and A, guanidinoacetic acid, citric acid, proline, valine, purine, hypoxanthine, nicotinic acid, fatty acids, steroids, diacylglycerol (14:0/0:0/8:0), imidazole, ornithine. ↓: Lysophophatidylcholine, putatively lysophophatidylethanolamine, docosahexaenoyl ethanolamide or tetradecanoylcarnitine, betaine aldehyde (++ vs –). 9 of these were in +- vs – and 12 in -+ vs –.	(43)
Plasma vs CSF in 20 high-grade (III and IV) gliomas and 11 controls	LC-MS targeted	Levels of most metabolites significantly correlated between plasma and CSF samples, with some only correlating in the control samples	(48)
40 paediatric medulloblastomas vs 11 controls	LC-MS untargeted (2x chromatography)	↑: S-Adenosyl-L-methionine, α-ketoglutarate, anserine, fumarate, hydroxypyruvate, malate, succinate, N-acetyl-aspartate, total triacylglycerols. ↓: 5-Oxo-D-proline, citrate, isocitrate, trans-aconitate, GABA, diacylglycerols, sphingomyelin, oxidised lipids.	(45)
31 glioblastomas (GBMs) vs 13 controls	LC-MS targeted	↑: Carnitine, 2-methylbutyrylcarnitine, shikimate, aminobutanal, uridine, N-acetylputrescine, farnesyl diphosphate (pre-treatment GBMs vs controls). ↑: Carnitine, propionylcarnitine, 2-methylbutyrylcarnitine, isobutyryl-L-carnitine, deoxycarnitine (*TP53*-wildtype GBM) ↑: Lactate, GABA, choline. (*TP53*-wildtype and *PTEN*-mutant GBMs)	(44)

↑ denotes increased metabolite abundance in disease vs control.

↓ denoted decreased metabolite abundance in disease vs control.

Since metabolites in extracellular spaces such as CSF are those excreted by cells, they may tell us inversely which metabolites are excessively consumed by the tumours, since tumours will take up what they need and excrete waste (35), see also (66). For example, a low level of glucose and high level of lactate in CSF indicates a high uptake and use of glucose by tumour cells, metabolising it though aerobic glycolysis for energy production. A low level of methionine indicates high methionine retention for cell growth. As the metabolites in CSF are not just from the tumour cells but from other brain and immune cells in the vicinity of the ventricles, they are not as specific as oncogenic ctDNA and relative levels of the metabolites in healthy and cancerous brain must be determined with thresholds rather than presence/absence (**Figure 2**) (34). One could say that CSF is useful for finding biomarkers but not for investigating the mechanism at the level of the tumour, for which tissue is necessary.

## 6 Challenges of using CSF circulating metabolites

The range of metabolite abundance within patients and within healthy individuals is wide due to diet, sex, age, genetics, medication and environment (**Figure 2**) (21, 35, 41, 44). The range measured can be widened still by differences in processing samples before storing at -80°C. The ranges in cancerous and healthy patients can therefore overlap, making a clear diagnosis of some patients difficult (41). Trying to match patients and controls, and generating strict SOPs for sampling will minimise these variables (41). It is important to use as biomarkers metabolites which are part of the “core” profile free from random daily noise factors (67). Metabolites are unlikely to vary by more than two-fold due to inter-individual variation and therefore it has been suggested that, in the absence of enough samples to undertake robust statistical analysis, a greater than two-fold fold change cut-off could be used to determine metabolites altered in abundance by disease (68). The median coefficient of variation of metabolite levels in that CSF cohort was 35%, which was lower than in plasma. The variation in another CSF cohort was about 15% for the majority of the metabolites; RSD < 30% for most compounds with medium abundance whilst it was common for the compounds that show high biological variation to be relatively less abundant (69). Therefore, inter-individual variation is not insurmountable when designing a CSF metabolome test for disease.

Another challenge in developing a clinical test for brain tumour diagnosis from CSF is the lack of healthy volunteer samples to compare to, because the samples are uncomfortable and risky to obtain compared to blood or urine samples and require highly trained medical staff [not trained science students (41)]. Therefore, most studies compare different brain tumours, primary to metastatic, or tumours to a different neurological disease. Nevertheless, these comparisons are also important to make sure the metabolic biomarkers are specific to a disease since common metabolic disturbances may exist across disorders (41).

As some brain tumours are rare, acquiring enough samples to have statistical power in a metabolomics experiment is challenging; most previous studies of the brain tumour CSF metabolome had low numbers of patients (less than 20 per group) apart from Lee and co-workers who had 40 medulloblastoma and 11 normal samples (45), and a couple of studies on brain metastases, which are the most common brain tumour (36, 60, 70). Therefore replication-validation of biomarkers in a second cohort enables low sample numbers to give reliable results (66, 71, 72). This second cohort data could come from re-analysing data collected by others, should data be made more widely available in central databases and repositories. Untargeted metabolomics data collected on different types of NMR spectrometer or mass spectrometer (even on different days for MS) cannot be combined for analysis as the acquisition of data affects the metabolite abundances and sensitivity, however they can be analysed as separate cohorts.

## 7 Methods for metabolite extraction from CSF and extract analysis

Methods for metabolite extraction (from CSF of brain tumour patients) vary from one research group to another, making it difficult to find an optimum method or a validated method (33, 34, 43, 45, 48, 57, 59–61). In one study, some optimisation of their protocol was undertaken and optimal solvents are methanol for extraction and 50% aqueous acetonitrile for reconstitution (60). Most methods involve adding methanol, regarding this as a way to precipitate out protein macromolecules, which would otherwise block the HPLC column. The methanol contact time varied considerably though, from the time it takes to vortex the samples (<1 minute) to overnight. When overnight, the temperature was low at -20 or -80°C, whereas for short periods room temperature or ice was acceptable. Sometimes chloroform is also used in a biphasic extraction. Alternate methods of protein removal include a molecular filter and sulfosalicylic acid. Where the method was for LC-MS and enough detail was given, the original volume of the sample was often restored upon evaporation and reconstitution, or else 12.5 times concentrated or 1/8th diluted. For GC-MS, samples have to be derivatised. Half of the studies were targeted metabolomics studies. Sampling site does not affect metabolite level because low molecular weight metabolites can diffuse rapidly through the CSF (57), unlike proteins (52). Therefore, the CSF does not need to be sampled from the ventricle adjacent to the tumour and all samples can be considered equally in future studies. The main instruments for CSF metabolome analysis in brain tumour research are mass spectrometer and NMR spectrometer.

NMR spectroscopy can measure CSF directly and is non-destructive (as the sample can be poured back out of the NMR tube and used for another analysis or stored) (41, 73). It measures all hydrogen-containing metabolites in a non-targeted manner, but they must be present at least at micromolar concentrations. The spectra aid structural identification and are quantitative.

Mass spectrometry can also measure CSF directly; however, it consumes the sample (as it gets ionised and inserted into the mass spectrometer and cannot be recovered) (41, 73). MS measures metabolites which can be ionised, and can measure at lower concentrations, thus covering a wider dynamic range. The best mass spectrometers for metabolomics are those that have high mass accuracy and resolution, since there are many isomeric metabolites and high resolution is needed to distinguish them. These mass spectrometers are Orbitrap and FT-ICR (74). The CSF metabolome database was partly created by putting neat CSF onto MS or LC-MS (75). A new ionisation technique was created for quickly measuring 1 µl neat CSF samples by MALDI-TOF/TOF; this could quantify glucose using an internal standard reproducibly (76). Generally though, direct infusion mass spectrometry is considered suitable for screening only (77). FT-ICR spectrometers can separate out groups of metabolites and stitch spectra back together, thus analysing metabolomics samples by direct infusion mass spectrometry (78). Ionisation suppression and matrix effects is the negative influence on the ionisation and the transfer of metabolites from liquid to gas phase, respectively, of other metabolites/salts also in the mass spectrometer at the same time (77). Therefore, in most cases, research groups use chromatography to separate out groups of metabolites by their physical properties into smaller packages (fractions) to enter the mass spectrometer, decreasing the number of metabolites at any time, increasing likelihood of low concentration- poor ionisers of being detected, and enabling use of retention time as extra means of identification when isomers exist (*e.g*. a good LC method will separate out leucine and isoleucine). Fragmentation mass spectra (MS^2^) aid in metabolite identification too. MS is not quantitative but with preceding chromatography, peak areas allow relative quantification (area in treatment divided by area in control) or absolute quantification (using internal standards and external calibration curves, which is only applicable to targeted studies of a few metabolites of interest).

Gas chromatography (GC) has high chromatographic resolution and GC-MS offers structural information from fragmentation (41, 73). Metabolites must be extracted from the CSF into a suitable solvent, and since only volatile molecules can pass through GC, they must be derivatised to increase the number of volatile metabolites, still not capturing the whole metabolome.

Liquid chromatography (LC) can detect a larger number of metabolites however the metabolites detected are biased by the solvent used for extraction and the column and mobile phase used for chromatography (41, 73). Examples of columns are normal phase and HILIC (hydrophilic interaction liquid chromatography) for polar metabolites and reversed phase for non-polar metabolites including lipids (77). Chromatographic resolution is not as high as GC and matrix effects mean the column has to be extensively pre-conditioned with your matrix (*e.g*. CSF) before your samples can be run, however thousands of metabolites can be detected in one sample.

Untargeted metabolomics experiments produce lists of 100s to 1000s of ions, most of which have likely molecular formulae determined and some of which are annotated with a metabolite name, determined by comparison to databases and libraries (41). To determine differences between sample groups (classes), unsupervised methods that do not know the classes of the samples (such as multivariate PCA) and supervised methods where the algorithm knows the class of each sample (such as univariate t-test, ANOVA, and multivariate OPLS-DA) are used. These tests produce shortlists of statistically significant metabolites, which can be mapped onto pathways and interpreted in the biological context.

## 8 Correlating tumour tissue or brain tissue with CSF metabolite profile

To the best of our knowledge, there has been no work published that correlates CSF metabolomic profile with brain tumour metabolomic profile, and only one paper that correlates CSF with brain tissue metabolomic profile from another neurological disease (namely tauopathies), so this is an open area of research. Although it is helpful that biomarkers specific to diseased patients can be found in CSF, it is only when tissue metabolites are extracted and CSF metabolites can also be found in tissue, that one knows they are markers of cancer and have a role in cancer’s pathogenesis (or other disease), rather than being byproducts of illness, the immune response, the gut microbiome or being drug-induced (17, 79–82). Targeted and untargeted metabolomic methods were used to analyse CSF, plasma and brain tissue from rats with and without tauopathies (66). When comparing the top twenty most discriminating metabolites from OPLS-DA in targeted analysis in each of the sample types, there was no overlap between those in plasma and brain tissue whereas the topmost discriminating metabolite in the CSF, thymidine, was the twentieth most discriminating metabolite in the brain tissue. This is a minimal amount of overlap and shows that the metabolites in the bodily fluids may be able to discriminate diseased from healthy people as biomarkers, but they do not give information on the underlying pathology of the disease. Three metabolites overlapped between CSF and plasma suggesting interchange of those metabolites. Where the level of a metabolite increased in CSF it also increased in plasma or tissue, and vice versa. Seven of the twenty most discriminating CSF metabolites in the pilot study were also in the top twenty CSF metabolites in the confirmation study cohort. Most interestingly, there was a trend towards increased myo-inositol abundance in brain tissue whereas significantly decreased myo-inositol abundance in CSF, in tauopathy rats, which the authors suggest may be due to increased consumption of myo-inositol by brain glial cells, depleting the CSF. In the untargeted analysis, CSF was discriminative of diseased and healthy rats, whereas tissue was not, even though more metabolites were detected in brain tissue than in CSF, showing the CSF metabolome to be a good biomarker. Creatinine level (significant) and phosphocreatine level (trend only), both breakdown products of creatine, were lowered in tauopathy rats in CSF and tissue respectively.

A few papers have compared previous research about tissue metabolomics to their CSF metabolomics results. For example, comparing three papers looking at three sample types (middle frontal cortex tissue, white matter tissue, and CSF), it was reported that there were higher levels of ceramides in the AD patients compared to normal controls in all three sample types (49). Also, having previously found energetic stress and mitochondrial dysfunction in brain tissue from three transgenic mouse models of familial AD, a team then found numerous pathways in CSF samples associated with energy metabolism and mitochondrial function, including the TCA cycle (most affected in mild cognitive impairment and AD) and saturated fatty acid metabolism (in AD), among other related pathways, corroborating the role for mitochondrial dysfunction in early AD (83). Therefore, systemic biofluids can yield meaningful information. Comparing the glioma metabolomics literature, we found no significant metabolites overlapped between studies comparing grade I or II and grade IV glioma tissue (24, 84) and studies comparing different grades of glioma CSF (34, 59). In particular the acids, citrate, lactate, pyruvate, and glycerate, were insignificant in tissue whereas they were among the few significant CSF metabolites. However, we could see overlap between the metabolites in the tissue grade comparisons and the metabolites significant between malignant glioma and control CSF samples (33). These included acetylcarnitine, myo-inositol, ribose phosphate (increase in grade 4 tumours, decrease in malignant glioma CSF), serine (increase in both), taurine (increase in both), proline (increase in both), and glutamine (decrease in tissue, increase in CSF). This shows that there is some overlap in metabolic signature between tissue and CSF samples but the difference (and often low number of significant metabolites in CSF comparisons) may be due to lower numbers of CSF samples used than tissue samples in typical papers, and so the statistical significance and reliability of biomarkers will be greatly increased with larger cohorts.

## 9 Correlation with magnetic resonance imaging and magnetic resonance spectroscopy

Locasale et al. investigated whether relative levels of individual metabolites in CSF of malignant glioma patients correlated with MRI measurements of tumour size (Tgad and FLAIR), and found six metabolites that correlated with both, four positively and two negatively (33). These were myo-inositol, cytidine, acetylcarnitine, acetoacetate, phenylpropiolic acid and cholesteryl sulfate. However, whereas metabolomics could cluster newly diagnosed and recurrent samples separately, MRI measurements were not able to draw out these clusters. CSF from leptomeningeal carcinomatosis (LC), a metastatic cancer invading the central nervous system (CNS), was compared to normal CSF and there was a clear metabolic profile difference in a study (36). When compared to MRI, the metabolic profile (either whole NMR spectrum or five biomarker metabolites) could be correlated with the grading of radiological leptomeningeal enhancement determined by MRI. CSF from patients with medulloblastoma with or without leptomeningeal metastasis were compared, and those with metastasis could be distinguished from those without by 27 low mass ions (9 of which could differentiate the group which was positive only on MRI scan [*i.e.* discordant cytology and MRI) from the group which was negative on both tests (*i.e.* without metastasis)] (43). The metabolome test could support MRI diagnostics rather than replace it, since false negatives and false positives have negative outcomes for children and two tests are usually used together to be certain of the metastatic diagnosis (MRI and CSF cytology). Nakamizo et al. investigated the CSF metabolite profiles of glioma patients by GC-MS and found that the metabolite profiles are not related to the tumour location and enhancement obtained using MRI (59). Additionally, MRS was performed for 9 of the patients’ tumours and there was correlation between CSF lactic acid concentration and ^1^H-MRS –determined tumour lactic acid level. However, although citric and isocitric acid were detected in CSF, they were not clearly detected by MRS. This suggests that the tumour metabolic condition, *i.e.* the pathways which are more and less active in the tumour cells, is reflected in the CSF.

In two studies outside of the CNS cancer field, firstly CSF metabolomics revealed dysregulated metabolic pathways which mapped to brain regions of mild cognitive impairment observed by structural MRI as clusters of contiguous voxels (85). Secondly, CSF metabolomics data was integrated with MRS metabolic data of the brain and found 3-hydroxyvalerate associated with frontal white matter N-acetyl aspartate levels (86).

## 10 Surveillance in drug treatment

Little has been published about the monitoring of drug effects on cancer using metabolomics, in the human body or animal models, as reflected by a 2021 review (87). For example in an animal model, the levels of seven metabolites in urine were perturbed by cancer but trended towards normal levels during drug treatment (64); in another animal model, lipid metabolism in tumour tissue was effected by a drug (88). Thirdly, in a human serum study, the effects of drug toxicity could be seen in lipid levels (89). Nevertheless, it stands to reason that effects drugs have on proteins would be reflected in the metabolic phenotype, and therefore this is a worthy avenue of research (87). Tumours that affect the central nervous system can be treated by drugs applied intraventricularly, a new method of application which is more direct and bypasses the BBB, therefore drug action could potentially be monitored in the CSF (90, 91). Some of those drugs used disrupt cancer metabolism, such as methotrexate, and the metabolic effects expected can be measured in the CSF (92). Monitoring can be done with less distress for the patient when serial samples can be taken through an Ommaya ventricular reservoir during treatment course. The concentration of drug could be quantified in parallel if requested. Applying methotrexate to a leukaemia patient resulted in less folates and S-adenosylmethionine and more homocysteine and adenosine in the CSF (92). Applying it to children with cancer resulted in more homocysteine, homocysteic acid and cysteine sulfinic acid, especially if they had neurologic toxicity from the methotrexate (65). When applying immunotherapy drugs to patients with malignant brain tumors the immune response to the drug could potentially be monitored by metabolic profiling because immune cells have a different metabolism when activated (73).

## 11 Evaluation of the tumour microenvironment

It has been stated that the majority of molecules in the CSF are released by other cells in the brain that surround the ventricles and only a small share from the tumour, therefore the CSF is mainly indicative of the tumour microenvironment (61). Nevertheless, tumour metabolites are detectable above the background, in altered abundances, as shown in **Table 1**. The tumour microenvironment could also be detected above the background. A joint analysis of the proteome, metabolome and lipidome aimed to use CSF to understand the recurrent medulloblastoma microenvironment (61). It showed anti-inflammatory and tumor-promoting proteins and hypoxia-associated proteins, metabolites induced upon hypoxia (whereas metabolites in pro-inflammatory pathways had the same low abundance in control and medulloblastoma samples), and an anti-inflammatory epoxygenase product and beta-oxidation promoting lipid hormone called 12,13-DiHOME, which were more abundant in medulloblastoma samples. Therefore, the microenvironment was able to be known and interpreted through multiomics, and without the proteome, the hypoxic nature of the metabolites and lipid would not have been able to be interpreted as such, due to the wide roles of metabolites. This signature was of an anti-inflammatory and pro-hypoxic microenvironment. The hypoxia-indicating metabolites were lysine, methionine, tryptophan and serine.

In another multiomics study of medulloblastoma CSF (where known, most were first occurrence, but the majority of sampling times were unknown), a joint analysis of the transcriptome, metabolome and lipidome indicated a hypoxic microenvironment, as total triacylglycerols lipid class accumulated and certain TCA cycle metabolites (α-ketoglutarate, fumarate, malate, and succinate) also accumulated (45). A reduction in glucose-derived acetyl-CoA due to hypoxia forces α-ketoglutarate to convert into citrate and then into acetyl-CoA for *de novo* lipogenesis. Genes representing TGF-β signaling, TNF-α signaling *via* NF-kB, and adipogenesis pathways were found. In multiomics integration, ubiquitin-fold modifier 1 (UFM1) was positively correlated with S-adenosyl-L-methionine and lysophosphatidylcholine 17:0 (LPC 17:0), though this is hard to biologically interpret with current knowledge.

A metabolomics study of glioblastoma (GBM) CSF associated higher levels of aminobutanal (an intermediate of GABA synthesis) and acetylcholine to worse overall survival, and these neurotransmitters suggest a link with surrounding brain tissue; it is known that GBM can increase acetylcholine receptor expression and use autocrine signaling to invade (44). The same paper found a bacterial metabolite, shikimate, increased in abundance in GBM CSF, and proposed the gut microbiome through the gut-brain axis, or hypothetically shikimate-producing bacteria in the CSF, affects GBM.

## 12 Stability of the CSF metabolome of the patient over time and of the CSF sample taken

### 12.1 Stability of patients’ metabolome over time

There is not much experimental information on whether samples of CSF from people or animals taken at different time points are stable in terms of the metabolome, nor whether the metabolome is even diurnally stable. The little information available on short term (less than 2 years) stability of the CSF metabolome, showed the same four metabolites distinguished Amyotrophic Lateral Sclerosis (ALS) from control, whether patient samples were taken at >6, >12 or >18 months post-baseline measurement in a longitudinal cohort, showing overall stability of the ALS CSF metabolome (93). Normal pressure hydrocephalus patients with a shunt had hourly CSF samples taken for analysis over a six-hour period to measure temporal changes in metabolome (94). Some patients undertook moderate exercise, which affected their metabolome at one hour post-exercise, with a lesser effect at three hours post-exercise, showing the effect is transient. Other patients did not exercise, and three metabolites reported show minimal variation over time compared to the variation induced by exercise. It must be noted that external lumbar drainage led to changes in the levels of two metabolites (lactate and 8-isoprostane) over 72 hours (significant after 48 hours) and so when placing and sampling from a shunt, the time point of sampling must be considered carefully and should be as close to initial shunt placing as possible (95). Looking at the longitudinal changes in the CSF metabolome of HIV patients on combined antiretroviral therapy, there was a difference in metabolome between the two CSF samples collected on two visits, for all groups of patients, even those with a stably impaired or normal neurocognitive function (96). There is no indication of the length of time between visits although it could have been roughly 6 months based on their previous work. This indicates that the human CSF metabolome is not steady during infection. Up to four longitudinal CSF samples were available for each participant in a study of AD risk factors amongst 1111 initially dementia-free middle-aged adults enriched for parental history of AD (50). The comparison of samples at different time points was not undertaken. In elderly adults (aged 73 years on average at first sampling), between two lumbar punctures taken four years apart, four metabolite levels in the kynurenine pathway did not change whereas quinolinic acid, kynurenine and 3-hydroxykynurenine levels increased over time, considered biomarkers of aging (97).

### 12.2 Stability of CSF samples

In terms of the stability of CSF samples taken, many more studies have been done. Samples of CSF, which had been centrifuged to remove cells and previously frozen at -80°C for a number of years, were taken for stability studies and metabolites were found to be stable for 72 hours at 5-8°C (*i.e.* on ice or in a fridge) or up to 8 h at 18–22°C (*i.e.* like room temperature) (98). A stability study on fresh CSF was undertaken with aliquots of samples stored either at room temperature for 2.5 hours (to mimic the time between collection and transfer to a fridge in the clinical laboratory), or on ice (~4°C) for 0.5, 2.5, 24 and 72 hours (to mimic storage in the lab fridge before sample processing, which could be same day, next day or after the weekend) (99). Samples were then centrifuged and frozen at -80°C. The study found no trends in CSF total metabolites’ levels with time and no gross differences in stability and all metabolites were similarly stable (apart from valine, which had slightly higher variation in abundance than the other metabolites assayed). Therefore, transit to the clinical laboratory at room temperature within 2.5 hours and storage in the fridge until processing is acceptable for samples destined for metabolomics. However, another study of delay times of 0.5 or 2 hours at room temperature before centrifugation and storage of CSF, showed increases in ten amino acids’ levels (for 6 the increase was significant only at 2 h) and 49 metabolites’ levels including glucose (for 25 the increase was significant only at 2 h), as assessed by targeted LC–MS amino acid analysis and untargeted GC-MS analysis respectively (100). This stresses the need to immediately cool and then centrifuge and snap freeze samples upon collection. The reason for this discrepancy in outcome between studies is unclear. In yet another study, levels of acetate, pyruvate and ascorbic acid changed greatly within 20 h of storage at room temperature even though the CSF samples had been optimally prepared in a pH stabilizing buffer (101). At lower temperature (4°C) in the same buffer these metabolites were stable.

## 13 Challenges or limitations of the clinical analysis of metabolites in CSF

The main bottleneck in untargeted metabolomics screening is metabolite identification, which, due to many isomers, needs a high resolution mass spectrometer, to distinguish species with very similar mass-to-charge ratio (*m/z*) and retention time to provide the exact mass, or a high frequency NMR spectrometer, to pick up low concentration metabolites and their molecular structures (102, 103). Metabolite identification with absolute certainty also needs multiple pieces of evidence, including matching to a standard (either *m/z* and retention time and MS/MS fragmentation spectrum, or ^1^H and ^13^C and 2D NMR peaks). Once the metabolite has been identified, the targeted analysis to determine sensitivity and specificity of the biomarker and threshold at which the biomarker becomes indicative of the cancer, can begin, for which there are no standard methodologies (43). If the sensitivity and specificity are not high enough then the analysis cannot be used in clinical practice because there will be too many false positives and false negatives (36). As the metabolites are likely not unique to the cancer, then high inter-patient variability means it is likely that a small number of future patients will fall outside of the indicative threshold determined by the targeted metabolomics (43). Translation to the clinic relies on the presence of analytical instruments in the hospital, preferably mass spectrometers capable of highly selective MS^n^ analysis of one or a few particular compounds, and the trained staff to undertake the analysis, so hospitals with neurooncology wards need to invest in such instruments (104). The risk is they are not keen to benefit from the technology. Taking the CSF sample for analysis in the lab is expected to be facile as the sample can be kept on ice or ice packs in transit and can be turned around quickly.

## 14 Future directions

In future, more work needs to be published on optimising a method for metabolome extraction from CSF as there is no indication of the optimal method for preparing samples for each analytical instrument, and an optimal method that all new researchers could use would expedite applied research. Indeed, the community needs to come to a consensus on the instrumental method it uses (NMR, LC, GC, MS), so that results can be compared between studies and a generic workflow for hospitals can be created. The method of centrifuging and freezing the CSF sample before analysis should be unified into a standard operating procedure (SOP) in order for a test to be reliably undertaken on patient samples and used by doctors. With elimination of differences in protocol, the results will be more uniform, only differing in biological variability, leading to clearer differences between groups when larger cohorts are recruited. Tests need to be quantitative and validated in larger test cohorts in order for them to be approved as diagnostics by health regulators. Mass spectrometers are currently in use in NHS hospitals in the UK so they can be used easily for these tests. In March 2022, the first multi-metabolite CSF assay of neuroinflammation to be clinically validated in an accredited pathology laboratory was published by an Australian team (105). This was based on groundwork of multiple human studies associating certain metabolic pathways with CNS inflammation. The LC-MS method is particularly useful in analysing one-off samples (as opposed to batched accrued) because you do not need to use special kits that need batch testing, can run one sample at a time on the LC-MS, and can rapidly turnaround the sample in 4 h (simple and rapid sample preparation) to get the data back to the doctor making the diagnosis. It is also cost-effective. This shows the potential for this type of assay to be developed and used clinically for CNS tumours in future.

Correlating tumour or brain tissue with CSF metabolite profile is a particularly important field of research to begin since it will show whether those metabolites seen in the CSF of patients with brain tumours actually reflect the brain tumour’s presence and grade/subgroup/aggressiveness or state of proliferation/migration/metastasis, or whether they simply reflect perturbation of the health of the individual and microenvironment. It is difficult to get hold of flash frozen brain tumour tissue for research so new methods for metabolite extraction from formalin-fixed paraffin-embedded (FFPE) tissue will help (106). Research into the stability of brain tumour patients’ CSF metabolomes over time, will tell us when samples should be taken for accurate diagnosis and comparison to peers’ samples. Correlation with MRI will show whether the test is accurate in longitudinal monitoring as well as whether it adds any earlier or more detailed information of cancer progression than MRI can provide. Further longitudinal monitoring of drug delivery will show whether that gives useful information to the clinic or is unimportant, as only two studies on methotrexate provide not enough information. In all areas much more research is needed to have more confidence in the CSF metabolomics method as a procedure for diagnosis, prognosis, and long-term monitoring of response to treatment in brain tumours. There are many brain tumours which have not yet been researched by this method including ependymoma, DIPG and oligodendroglioma, so there is much scope for more researchers to enter this field. Overall, we can see that CSF monitoring by metabolomics has many potential uses and advantages over other tests but needs more research investment in order to become a reality.
